# DENTAL-RELATED DECISION-MAKING CAPACITY IN OLDER ADULTS WITH VARIED COGNITIVE FUNCTIONS

**DOI:** 10.1093/geroni/igad104.2097

**Published:** 2023-12-21

**Authors:** Xi Chen, Jirakate Madiloggovit-Lower, Carissa Comnick

**Affiliations:** The Ohio State University, Columbus, Ohio, United States; Thammasat University, Klong Laung, Pathum Thani, Thailand; University of Iowa College of Dentistry, Iowa City, Iowa, United States

## Abstract

**Background:**

Decision-making is often impaired in individuals with cognitive impairments. However, how cognitive impairment affects dental treatment decision-making remains unclear. We sought to describe dental-related decision-making capacity in older adults with varied cognitive functions.

**Methods:**

Sixty-five participants, including 14 healthy controls and 51 with documented cognitive impairment, were categorized into healthy controls, mild cognitive impairment (MCI), mild dementia, moderate dementia and severe dementia. A hyperthetical dental problem along with three treatment options were presented. Participants were first asked to identify the problem, related treatment options and the pros and cons of each option. They were then asked to choose a treatment using the given funds. Visual and verbal cues were provided if participants failed to complete the task independently. Participants were scored based on their performances. Data was analyzed using the Kruskal- Wallis test and Holm method for multiple comparisons and a linear regression model for the multivariate analysis.

**Results:**

Ninety-six percent of the participants with cognitive impairment showed some sorts of deficits in treatment decision-making. Sixty percent of those with MCI and 70% of participants with mild dementia could not explain pros and cons of the treatment choices, significantly higher than that in the healthy controls (10%). The decision-making capacity significantly differed across groups (p< 000.1). Compared to the health controls, decision-making capacity decreased 18%, 33%, 71% and 86% in the MCI, mild dementia, moderate dementia and severe dementia groups, respectively.

**Conclusion:**

Dental-related decision-making deficits was highly prevalent in older adults with cognitive impairment and occurred as early as MCI.

